# Pleural Effusion and Chylothorax in Congenital Diaphragmatic Hernia—Risk Factors, Management and Outcome

**DOI:** 10.3390/jcm13061764

**Published:** 2024-03-19

**Authors:** Yannick Schreiner, Sidre Sahin, Christiane Otto, Meike Weis, Svetlana Hetjens, Kathrin Zahn, Michael Boettcher, Alba Perez Ortiz, Neysan Rafat

**Affiliations:** 1 Department of Neonatology, University Children’s Hospital Mannheim, University of Heidelberg, 68167 Mannheim, Germany; yannickalexander.schreiner@umm.de (Y.S.); alba.perez-ortiz@umm.de (A.P.O.); 2 Department of Anesthesiology and Critical Care Medicine, University Medical Centre Manheim, University of Heidelberg, 68167 Mannheim, Germany; 3 Department of Gynecology and Obstetrics, University Hospital Mannheim, University of Heidelberg, 68167 Mannheim, Germany; christiane.otto@umm.de; 4 Department of Clinical Radiology and Nuclear Medicine, University Medical Center Mannheim, University of Heidelberg, 68167 Mannheim, Germany; meike.weis@umm.de; 5 Department of Medical Statistics and Biomathematics, Medical Faculty Mannheim, University of Heidelberg, 68167 Mannheim, Germany; svetlana.hetjens@medma.uni-heidelberg.de; 6 Department of Pediatric Surgery, University Children’s Hospital Mannheim, University of Heidelberg, 68167 Mannheim, Germany; kathrin.zahn@umm.de (K.Z.); michael.boettcher@umm.de (M.B.); 7 Department of Neonatology, Center for Children, Adolescent and Women’s Medicine, Olgahospital, Klinikum Stuttgart, 70174 Stuttgart, Germany

**Keywords:** CDH, chylothorax, pleural effusion, chest tube, ECMO, chronic lung disease

## Abstract

**Background:** Pleural effusion and chylothorax are common complications in the treatment of congenital diaphragmatic hernia (CDH). We set out to identify risk factors for chylothorax development in patients with CDH and to investigate the association of pleural effusion and chylothorax with neonatal morbidity and mortality. **Methods:** In this retrospective cohort study, we included 396 neonates with CDH treated at our institution between January 2013 and June 2019. Preoperative and postoperative chest radiographs and clinical data were evaluated and correlated with morbidity, complications and mortality. **Results:** Laboratory-confirmed chylothorax occurred in 58 (18.6%) of all CDH cases. Pleural effusion was frequently observed as a postoperative complication but also occurred as a pre-existing condition. Neonates with large defects of size C and D, patch repair, the need for presurgical and/or postsurgical ECMO support, pulmonary hypertension, liver-up phenomenon and lower relative fetal lung volume were associated with higher occurrences of chylothorax. After stepwise logistic regression, larger CDH defects (*p* < 0.0001) and the need for postsurgical ECMO (*p* = 0.0158) remained significant risk factors for CTX to occur (AUC 0.71). The same potential risk factors were used to assess their association with both presurgical and postsurgical pleural effusion. After stepwise logistic regression, only the need for presurgical ECMO remained significantly associated with presurgical PE (*p* < 0.01, AUC 0.65) and patch repair as the therapeutic intervention remained significantly associated with the occurrence of postsurgical PE (*p* < 0.0001, AUC 0.80). Patients with CTX had longer durations of both MV (*p* < 0.0001) and subsequent ventilatory assistance with spontaneous breathing (*p* = 0.0004), increased total lengths of hospitalization (*p* < 0.0001), increased durations of ECMO (*p* < 0.01) and increased incidences of CLD (*p* < 0.0001) compared to patients without CTX. No significant difference could be found for survival in both groups (*p* = 0.12). **Conclusions:** Our data suggest that the incidence of chylothorax is associated with large diaphragmatic defects, the need for postsurgical ECMO and the development of chronic lung disease, but not with survival.

## 1. Introduction

In congenital diaphragmatic hernia (CDH), a tissue defect in the developing diaphragm allows visceral organs to protrude into the thoracic cavity, thus impairing lung and heart development prenatally. As a result, neonates with CDH can present at birth with respiratory failure and severe pulmonary hypertension (PHT), leading to right heart failure. Once born, the ultimate approach to treat affected children is the reposition of the herniated organs followed by surgical repair of the diaphragm by either primary closure or patch interposition. In severe cases of CDH, extracorporeal membrane oxygenation (ECMO) is needed for stabilization and to overcome PHT to tolerate definitive surgery. After surgical repair, both pleural effusions (PEs) and chylothorax (CTX), which in most cases had not been pre-existent, occur solely as a complication of the intervention. Their underlying cause can be due to the iatrogenic damage of lymph vessels within the field of operation [1] or simply wound secretion and effusion due to the manipulation in the operated field. There are numerous reports on CTX after CDH surgery [1,2,3], but it must also be considered that some CTX might even occur presurgically. This might happen as a result of either herniated organs leading to the compression of lymph vessels or the vena cava, thus increasing intravascular pressure, leading to the capillary leakage of chylus, or as a sign of right heart failure most likely resulting from pulmonary hypertension of the newborn. Right heart failure is also believed to be the most common reason for pleural effusions in the absence of any intervention. Some, but not all, PEs and CTX become clinically relevant as they can impair respiration. Until now, it has not been clear if and when PEs in neonates with CDH should be treated by the insertion of chest tubes. Currently, this issue is controversially discussed, as chest tubes can lead to the overexpansion of the ipsilateral hypoplastic lung and to a thoracic midline shift, thereby potentially causing pneumothorax or PE on the opposite side, which may contribute to adverse outcomes [4]. We aim to identify risk factors which are associated with the development of CTX in CDH and to investigate the association of PE with and without chest tube insertion and CTX with children’s morbidity and mortality.

## 2. Materials and Methods

### 2.1. Study Cohort

In this retrospective cohort study, all neonates with CDH who were treated at the Department of Neonatology of University Medical Center Mannheim of the University of Heidelberg between 01/2013 and 06/2019 were analyzed. Exclusion criteria were gestational age < 34 weeks, age > 28 days when diagnosed or transferred to our center, associated anomalies, syndromes and chromosomal aberrations. Also, neonates with congenital heart defects were ruled out. Clinical data including pre- and postnatal parameters were collected. Incidences of chylothorax, pleural effusions requiring chest tube insertion and their flowrates were monitored both before and after surgery. The CDH Study Group Staging System [5] was used to define the defect size of the diaphragm. Concerning therapeutical interventions, the duration of ECMO and the application of diuretics and somatostatin were assessed. As outcome parameters, the duration of mechanical ventilation (MV) and subsequent ventilatory assistance with spontaneous breathing, duration of hospitalization, incidence of chronic lung disease (CLD) and its severity and survival were analyzed. This study was approved by the local ethics committee of the Medical Faculty Mannheim of the University of Heidelberg (reference number: 2019-827R, approval date 4 April 2019).

### 2.2. Diagnosis and Treatment of CLD, Chylothorax and Pleural Effusion

The diagnosis of CLD was performed as reported before [6,7]: if there was an additional need for oxygen supplementation on day 28 after birth, CLD was diagnosed. The severity of CLD was differentiated into three grades according to the additional need for oxygenation on day 56 after birth: mild CLD with no need for supplemental inspired oxygen (fraction of inspired oxygen (FiO_2_) ≤ 0.21), moderate CLD (FiO_2_ 0.22–0.29) and severe CLD (FiO_2_ ≥ 0.30). Chylothorax was defined by elevated triglycerides > 100 mg/dL and cell count > 1000 per µL predominantly showing lymphocytic differentiation (>50%) in drained effusion fluid and primarily treated conservatively. If chylus flowrates exceeded 30 mL/kg body weight/day, somatostatin administration via continuous infusion was initiated according to the following protocol: 5 µg/kg/h on day 1, 7.5 µg/kg/h on day 2, 10 µg/kg/h on day 3 and ongoing for 14 to 21 days depending on persisting chylus flow rates. Chest tube insertion to treat PEs was not routinely performed during or after surgery. To assess whether chest tubes were required during the postoperative period, the effusion volumes detected via ultrasound, chest radiography and blood gas analysis were taken into consideration. On a chest X-ray, a PE must have led to the collapse of the ipsilateral lung, thus leading to separation from the thoracic wall and/or to midline shift accompanied by the impaired ventilation of oxygenation for chest tube insertion to be performed. Once put in, chest tubes were kept in place for another two days after their flowrate declined to a minimum.

### 2.3. Data Analysis

Categorical variables are presented as percentages, whereas continuous variables are presented as either means ± standard deviation (SD). To compare groups regarding qualitative parameters, a Chi-square test or Fisher’s exact test was used, where appropriate. The mean values of the two groups were compared by two-sample *t*-tests (in the case of normally distributed data) or the Mann–Whitney U-test. The mean values of more than two groups were compared by ANOVA (in the case of normally distributed data) or the Kruskal–Wallis test, and Bonferroni correction was carried out. Regarding the incidence of chylothorax, CDH defect size (A + B = smaller defects, C + D = bigger defects), the type of performed surgery (patch interposition vs. primary closure), the presurgical and postsurgical need for ECMO (yes vs. no) and the incidences of severe pulmonary hypertension and the liver-up phenomenon were investigated as potential risk factors. Once odds ratios and 95% confidence intervals were calculated for each risk factor individually, stepwise logistic regression analyses were performed to adjust potential risk factors for each other. A receiver operating characteristic (ROC) curve and the area under the curve (AUC) were calculated to assess the scientific merit of the model. A *p*-value < 0.05 was considered statistically significant. SAS software version 9.4 (SAS Institute Inc., Cary, NC, USA) was used throughout the statistical analysis.

## 3. Results

### 3.1. Demographic and Clinical Characteristics of the Study Cohort

**Figure 1:**
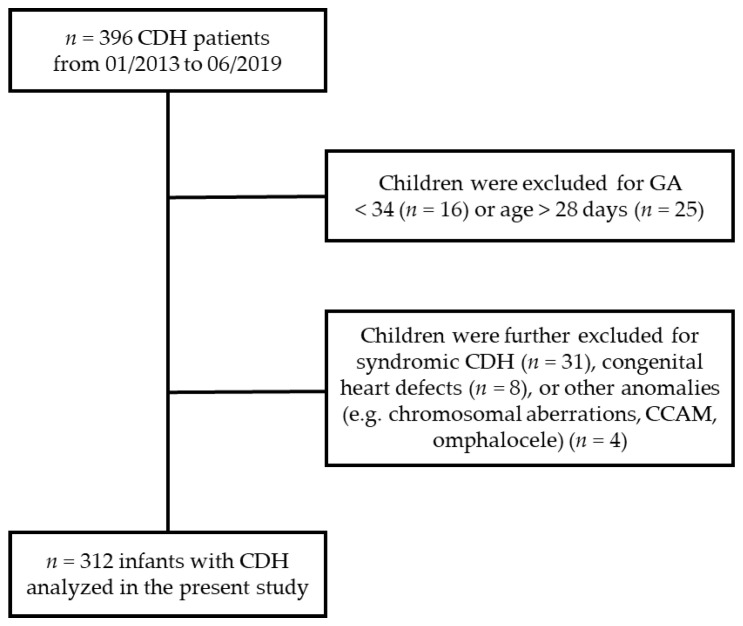
Flow chart representing the composition of the study population.

Between January 2013 and June 2019, 396 neonates with CDH were treated at our center, of which, 312 neonates were included in this study. For an overview of the recruitment of the study population and the characteristics of the dropouts, please see Figure 1. The enrolled study population consisted of 266 patients (85.3%) with left-sided and 46 patients (14.7%) with right-sided CDH. Chylothorax occurred in 58 (18.6%) of all CDH cases, 20.3% in LCDH and 8.70% in RCDH (*p* = 0.07). PE was frequently observed as a postsurgery complication (71.5%) but did also occur as a pre-existing condition. Overall survival was 82% for LCDH and 85% for RCDH, respectively. For a more detailed overview of the characteristics of the study population, please refer to Supplemental Table S1.

### 3.2. Risk Factors Associated with Developing Chylothorax and Pre- or Postsurgical Pleural Effusion

In the univariate analysis, among the investigated parameters, smaller diaphragmatic defects (A and B defect size according to the CDH Study Group Consensus) were less frequently associated with chylothorax when compared to larger defects of size C and D (OR 0.19, 95%CI 0.09–0.42, *p* < 0.0001). Neonates who underwent patch repair had a higher occurrence than those undergoing primary closure of the diaphragm defect (OR 6.52, 95%CI 1.96–21.7, *p* = 0.0006). Furthermore, the need for presurgical (OR 4.04, 95%CI 2.02–8.10, *p* < 0.0001) and/or postsurgical (OR 11.6, 95%CI 2.27–59.1, *p* = 0.0019) ECMO support was associated with the higher occurrence of chylothorax compared to patients who did not require ECMO. Also, both severe pulmonary hypertension and the liver-up phenomenon did show a significant association with the development of CTX (OR 4.30, 95%CI 1.86–9.91, *p* < 0.001 and OR 0.16, 95%CI 0.07–0.37, *p* < 0.0001, respectively). Concerning prenatal prognosis parameters for CDH, only lower relative fetal lung volume (rFLV) was associated with the occurrence of chylothorax (OR 0.95, 95%CI 0.92–0.98, *p* < 0.01) and a lower observed-to-expected lung-to-head-ratio (o/e LHR) was not (OR 0.98, 95%CI 0.95–1.01, *p* = 0.13). However, after stepwise logistic regression, larger CDH defects (*p* < 0.0001) and the need for postsurgical ECMO (*p* = 0.0158) remained significant risk factors for CTX to occur (AUC 0.71).

The same potential risk factors were used to assess their association with both presurgical and postsurgical pleural effusion. In the univariate analysis concerning presurgical PE, smaller diaphragmatic defects were less frequently associated with presurgical PE when compared to larger defects (OR 0.26, 95%CI 0.12–0.59, *p* < 0.001). Furthermore, the need for presurgical (OR 6.02, 95%CI 3.12–11.6, *p* < 0.0001) ECMO support was associated with the higher occurrence of presurgical PE, while the need for postsurgical ECMO support was not (OR 2.52, 95%CI 0.45–14.2, *p* = 0.27). Also, both severe pulmonary hypertension and the liver-up phenomenon did show a significant association with the development of presurgical PE (OR 6.47, 95%CI 2.49–16.8, *p* < 0.0001 and OR 0.40, 95%CI 0.19–0.84, *p* < 0.05, respectively). Concerning prenatal prognosis parameters for CDH, neither lower rFLV nor lower o/e LHR values were significantly associated with the occurrence of presurgical PE (OR 0.99, 95%CI 0.96–1.02, *p* = 0.42 and OR 0.98, 95%CI 0.94–1.00, *p* = 0.12, respectively).

In the univariate analysis concerning postsurgical PE, smaller diaphragmatic defects were less frequently associated with postsurgical PE when compared to larger defects (OR 0.06, 95%CI 0.02–0.19, *p* < 0.001). Postsurgical PE occurred more often in patch repair approaches vs. interventions by primary closure (OR 16.5, 95%CI 8.14–33.5, *p* < 0.0001). Furthermore, the need for presurgical (OR 11.7, 95%CI 4.10–33.6, *p* < 0.0001) ECMO support was associated with the higher occurrence of postsurgical PE, while the need for postsurgical ECMO support was not (OR 2.23, 95%CI 0.28–18.0, *p* = 0.69). Also, both severe pulmonary hypertension and the liver-up phenomenon did show a significant association with the development of postsurgical PE (OR 5.69, 95%CI 3.00–10.8, *p* < 0.0001 and OR 0.14, 95%CI 0.07–0.28, *p* < 0.0001, respectively). Concerning prenatal prognosis parameters for CDH, both lower rFLV and lower o/e LHR values were associated with the occurrence of postsurgical PE (OR 0.96, 95%CI 0.93–0.99, *p* < 0.01 and OR 0.95, 95%CI 0.91–0.99, *p* ≤ 0.01, respectively).

After stepwise logistic regression, only the need for presurgical ECMO remained significantly associated with presurgical PE (*p* < 0.01, AUC 0.65) and patch repair as the therapeutic intervention remained significantly associated with the occurrence of postsurgical PE (*p* < 0.0001, AUC 0.80).

### 3.3. Chylothorax Is Associated with Severe Morbidity but Not Mortality

**Figure 2:**
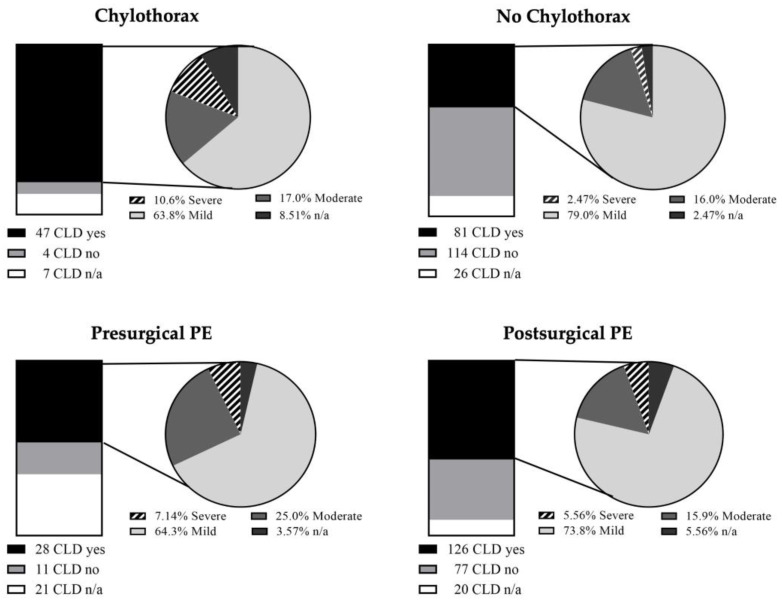
Incidences and severity of chronic lung disease (CLD) in children with chylothorax and pleural effusions (PEs). In children with chylothorax, CLD was frequently observed (47/58) and happened to be severe in 10.6% of all CLD cases. In children without chylothorax, both CLD in general (81/221) and severe CLD in particular (2.47%) were less frequent conditions (*p* < 0.0001). For presurgical PE (*n* = 60) and postsurgical PE (*n* = 223), CLD occurred in 28 (46.7%) and 126 (56.5%) cases, respectively. Both were significantly increased when compared to the controls exhibiting no presurgical or postsurgical PE.

To assess morbidity and mortality among patients with CTX, the study population was divided into two groups. Group 1 did exhibit CTX and Group 2 did not (including patients exhibiting PE but no CTX) (Supplemental Table S2). CTX occurred in 58 children (18.6%) in the study cohort (Supplemental Table S2). Patients with CTX exhibited significantly longer durations of both MV (*p* < 0.0001) and subsequent ventilatory assistance with spontaneous breathing (*p* = 0.0004) (Supplemental Table S2). Consistently, the total length of hospitalization was significantly higher in children with CTX (*p* < 0.0001) (Supplemental Table S2). Furthermore, an increased duration of ECMO (*p* < 0.01) was significantly associated with the presence of CTX (Supplemental Table S2). Also, CLD was more frequently present in patients with chylothorax compared to those without the condition (81.0% vs. 36.2%; *p* < 0.0001). The incidences of CLD and the distribution of severity scores are shown in Figure 2. With regard to survival, no significant difference could be found for both groups (*p* = 0.12) (Supplemental Table S2).

**Figure 3:**
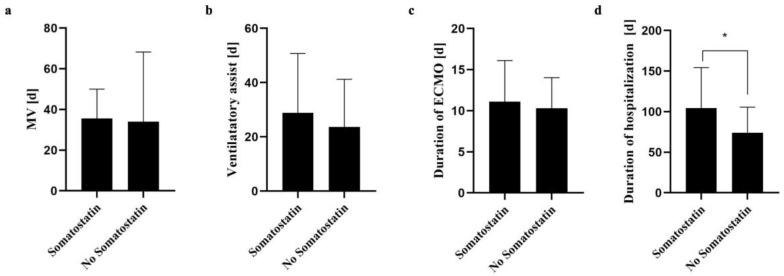
Comparison of outcome parameters of children undergoing somatostatin therapy of chylothorax. No significant differences could be determined regarding the duration of mechanical ventilation (MV) (**a**), the duration of subsequent ventilatory assistance with spontaneous breathing (**b**) or extracorporeal membrane oxygenation (ECMO) (**c**). Somatostatin therapy significantly increased the total duration of hospitalization of survivors (**d**). Data are presented in means ± SD. * *p* < 0.05.

Thirty-four patients received somatostatin at a mean duration of 21.4 ± 8.4 days when chylus flowrates exceeded 30 mL/kg body weight/day, which was associated with a significantly increased duration of hospitalization (Figure 3d). The durations of MV, ventilatory assistance with spontaneous breathing and ECMO were not associated significantly with somatostatin administration (Figure 3a–c).

### 3.4. Pleural Effusion Frequently Accompanies CDH and Is Associated with ECMO Use

Pleural effusions were observed to occur both pre- and postsurgically. In children with LCDH, presurgery PE occurred in 48 cases (18.0%), of which, 16 (33.3%) were ipsilateral, 20 (41.7%) were contralateral and 10 (20.8%) occurred bilaterally (Supplemental Table S3). For RCDH, PE prior to surgery occurred in 12 cases (26.1%), of which, 4 (33.3%) were ipsilateral, 4 (33.3%) were contralateral and 3 (25.0%) were located on both sides (Supplemental Table S3). There was no significant difference concerning the frequency of PE depending on the side of CDH (*p* = 0.09). However, in only 19 (39.6%) of LCDH and 5 (41.7%) of RCDH cases, chest tube insertion was indicated even before the surgical repair of the diaphragm defect. If so, the mean drained volume was 449 ± 508 mL. On the other hand, postsurgical PE was observed more frequently. For LCDH, 192 (72.2%) post-interventional PEs were registered, most of which (*n* = 164 (85.4%)) occurred ipsilaterally; for RCDH, 31 (67.4%) PEs occurred after surgery, of which, 27 (87.1%) also predominantly occurred ipsilaterally (Supplemental Table S3).

In 58.9% of postsurgical effusions in LCDH and 58.1% of those in RCDH cases, chest tube insertion was required during the postoperative period (Supplemental Table S3). If so, the mean drained volume was 704 ± 1161 mL. Supplemental Table S3 provides a detailed overview about incidences of PEs and the need for chest tubes during the preoperative and postoperative period.

The need of ECMO before surgery was significantly associated with presurgical and postsurgical PEs (*p* < 0.0001). Also, chest tube insertion was significantly more often required in this subset of patients (*p* < 0.05). Concerning children’s outcomes, both CLD (*p* < 0.0001) and major cerebral complications (*p* < 0.0001) such as cerebral atrophy, intracerebral hemorrhage and stroke were significantly associated with ECMO application.

### 3.5. Pre- and Postsurgical Pleural Effusion Is Associated with Clinical Outcome

**Figure 4:**
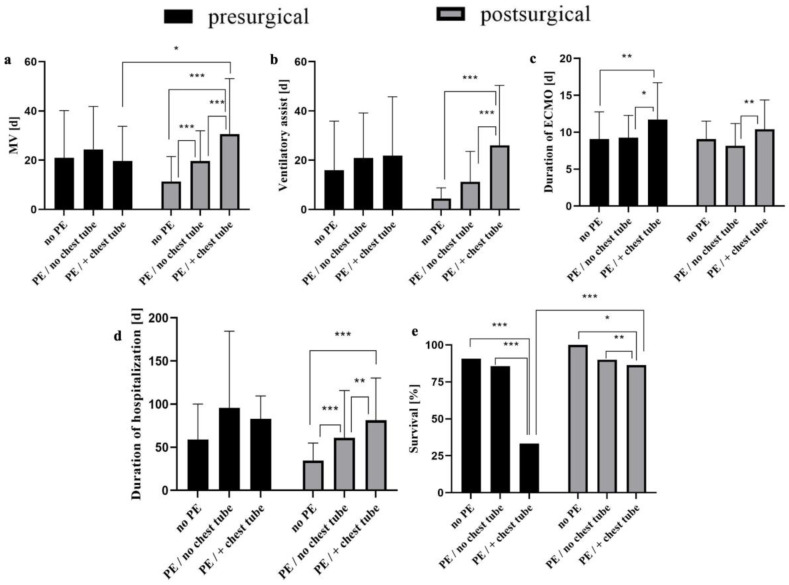
Outcome parameters of children with and without presurgical or postsurgical effusions (Pes) and with and without chest tube insertion, respectively. Duration of mechanical ventilation (MV) (**a**), subsequent ventilatory assistance with spontaneous breathing (**b**), duration of ECMO (**c**), duration of hospitalization of survivors (**d**) and survival (**e**) were measured and compared according to the presence of PE or chest tubes, respectively. Data are presented in means ± SD. Survival is given as percentages based on the respective study group population. * *p* < 0.05, ** *p* < 0.01, *** *p* < 0.0001.

High-volume presurgical PE requiring chest tube insertion was not associated with the duration of MV (Figure 4a), nor the length of ventilatory assistance with spontaneous breathing (Figure 4b) or the duration of hospitalization (Figure 4d). But in this subset of patients, increased ECMO duration (Figure 4c) was associated with poorer survival (Figure 4e). On the other hand, postsurgical PEs were significantly associated with the increased duration of MV (Figure 4a), ventilatory assistance with spontaneous breathing (Figure 4b) and the duration of both ECMO (Figure 4c) and hospitalization (Figure 4d). Likewise, a small but significant association with decreased survival could be determined (Figure 4e). Both presurgical and postsurgical PEs were associated with higher incidences of CLD (*p* = 0.008 for presurgical PE, *p* < 0.0001 for postsurgical PE).

## 4. Discussion

In the present study, we evaluated the incidence of PE and CTX, their risk factors and their impact on morbidity and mortality in CDH patients. Large diaphragmatic defects and postsurgical ECMO treatment are associated with an increased incidence of CTX. Presurgical ECMO is associated with an increased incidence of presurgical PE, and patch repair is associated with an increased incidence of postsurgical PE. Furthermore, CTX incidence is associated with an increased duration of MV and ventilatory assistance with spontaneous breathing, total length of hospitalization, duration of ECMO and incidence of CLD, but not survival. Yet, survival amongst patients with high-volume presurgical PEs requiring chest tube insertion is poorest. The incidence of postsurgical PE is associated with an increased duration of MV and ventilatory assistance with spontaneous breathing, duration of ECMO and hospitalization, incidence of CLD and poorer survival.

To date, the incidence of CTX after CDH repair remains uncertain and ranges between 5 and 27% [8]. Since not every PE needs to be drained via a chest tube and due to the lack of uniform diagnostic criteria, the incidence of CTX is most probably underestimated. Furthermore, as we report an incidence of roughly 19% with CTX predominantly occurring in cases of patch repair, the occurrence of chylothorax might mainly depend on the preferred surgical procedure to treat CDH and the size of the defect. Our results concerning risk factors for CTX are in line with results of previous studies which have already identified ECMO treatment and patch repair as risk factors for the development of CTX in univariate analysis [9,10,11]. Contradictorily, Heiwegen et al. could not identify any significant risk factors associated with CTX after adjusting risk factors for each other within the regression model, with none remaining significant [12]. Gonzalez et al. also reported on prolonged duration of MV and total length of hospitalization in 10 CTX patients even though survival did also not differ significantly [9]. We confirm these results after multivariate regression analyses in a much higher study population.

We were unable to examine the association of somatostatin therapy on its different underlying causes. As Beghetti et al. demonstrated before, CTX evolving for traumatic reasons as, e.g., in thoracic surgery occurs earlier than in cases with elevated central venous pressure and responds best to conservative therapy [13]. As it was not necessary in our study population to perform any surgical procedure for CTX to be resolved, we would assume retrospectively that most of our reported CTX cases occurred as a postsurgical complication.

Chylothorax is primarily treated conservatively, with either medium-chain triglycerides via enteral feeding or total parenteral nutrition being the first therapeutic approach to reduce chylus production [14]. If there is no sufficient response by this, somatostatin or its analogs should be considered. Somatostatin and octreotide were shown to be effective in resolving chylothorax in different clinical trials in adults [15] and infants [16,17,18] by reducing splanchnic blood flow [19] and the intestinal secretion of electrolytes and water [20]. In our study, somatostatin therapy was initiated if the chylus flowrate exceeded 30 mL/kg body weight/day, upon which, the total length of hospitalization increased, but neither of the other outcome parameters changed significantly. Therefore, despite the potential success of somatostatin reducing CTX flowrates and PE volumes, the clinical benefit of somatostatin therapy on outcome parameters is questionable. Moreover, we observed a significantly higher incidence of pneumonia in our study population with CTX, probably also promoted by the increased duration of MV. However, if chylothorax remains unresponsive to conservative treatment strategies, surgical approaches might be considered. Thoracoscopic ligations of chylus leakage were already successfully performed in several children who suffered from chylothorax for various reasons (postoperative vs. congenital), including CDH [21].

The rapid overexpansion of the lungs due to the insertion of chest tubes can represent a significant cause for morbidity and mortality in CDH [4]. Via strong suction and increasing vacuum, midline shift and contralateral pneumothorax are possible consequences contributing to adverse outcome. In our study, even though PEs were frequently observed during the postoperative period when CDH was corrected, chest tube insertion was only necessary in 59% of all PEs occurring postoperatively. A routine insertion of chest tubes during surgical CDH repair might therefore not be necessary and only performed postoperatively when clinical indications are present. The higher incidence of CLD in this cohort might likely result from the duration of MV and ventilatory assistance, both being increased depending on the effusion volume.

Presurgical PE, on the other hand, was less frequently observed, and although it did not alter the selected outcome parameters, CLD was determined more frequently if PEs were present and survival was poorest when high-volume presurgical effusions occurred. Therefore, presurgical PE requiring chest tube insertion is associated with high mortality. Hence, in this population, the duration of MV and ventilatory assistance with spontaneous breathing would probably increase tremendously if they survived longer. This bias might account for the fact that we could not determine significant changes in our outcome parameters for presurgical PE despite lower survival rates in cases when chest tubes were required. Our study has several limitations: it is a retrospective, observational, single-center study with a limited sample size. We only included children with isolated CDH and GA > 34 gestational weeks into our analysis to avoid bias. Therefore, PE and CTX and their association with morbidity and mortality has not been evaluated for children with multiple malformations or patients born preterm (GA < 34). The use of ultrasound or chest X-ray in detecting PE might introduce variations and bias. In our study, chest X-ray was first performed to detect PE, and ultrasound was subsequently used to quantify PE. A comparison of both approaches was not performed. A prospective multicenter approach with a greater number of patients should be performed in order to identify related characteristics for the development of PE and CTX and their contribution to morbidity and mortality.

## 5. Conclusions

Chylothorax is associated with large diaphragmatic defects and postsurgical ECMO treatment, while postsurgical PE is associated with patch repair. Furthermore, CTX in CDH is associated with the increased duration of MV and ventilatory assistance with spontaneous breathing, total length of hospitalization, duration of ECMO and incidence of CLD, but not survival. Yet, survival amongst patients with high-volume presurgical PEs requiring chest tube insertion is poorest. The incidence of postsurgical PE is associated with the increased duration of MV and ventilatory assistance with spontaneous breathing, duration of ECMO and hospitalization, incidence of CLD and poorer survival.

## Data Availability

Data are contained within the article.

## Supplementary Materials

The following supporting information can be downloaded at: https://www.mdpi.com/article/10.3390/jcm13061764/s1, Table S1: Characterization of the study population; Table S2: Comparison of outcome parameters for chylothorax vs. no chylothorax; Table S3: Frequency of pleural effusion and drainage before and after surgery for left- and right-sided congenital diaphragmatic hernia.
