# Supplementary material for: Pleural Effusion and Chylothorax in Congenital Diaphragmatic Hernia—Risk Factors, Management and Outcome
Source: J Clin Med. 2024 Mar 19;13(6):1764. doi: 10.3390/jcm13061764 (PMC10971182; doi:10.3390/jcm13061764)
Supplement: Supplementary file 1 [file jcm-13-01764-s001.zip › Supplemental Table S1.pdf]

**Supplemental Table S1: Characterization of the study population.**

| | LCDH, *n (%)* | | RCDH, *n (%)* | |
|---|---|---|---|---|
| **Total** | **266** | **(100)** | **46** | **(100)** |
| Sex male | 157 | (59.0) | 24 | (52.2) |
| Inborn | 221 | (83.1) | 36 | (78.3) |
| Prenatally detected | 229 | (86.1) | 36 | (78.3) |
| Polyhydramnion | 72 | (27.1) | 19 | (41.3) |
| FETO | 10 | (3.76) | 2 | (4.35) |
| o/e LHR [%], *Means ± SD* | 39.3 | ± 12.8 | 44.3 | ± 13.2 |
| rFLV [%], *Means ± SD* | 36.7 | ± 16.0 | 29.6 | ± 14.8 |
| Chylothorax | 54 | (20.3) | 4 | (8.70) |
| ECMO prior to surgery | 106 | (39.8) | 31 | (67.4) |
| ECMO post-surgery | 8 | (3.01) | 2 | (4.35) |
| Mechanical ventilation | 234 | (88.0) | 42 | (91.3) |
| Gestational weight [g], *Means ± SD* | 3061 | ± 508 | 3058 | ± 502 |
| 5-minute APGAR, *Means ± SD* | 7.51 | ± 1.60 | 7.20 | ± 1.55 |
| **CDH characteristics** | | | | |
| Upside stomach | 164 | (61.7) | 0 | (0) |
| Upside gut | 228 | (85.7) | 34 | (73.9) |
| Upside liver | 136 | (51.1) | 37 | (80.4) |
| Upside kidney | 7 | (2.63) | 0 | (0) |
| Upside spleen | 208 | (78.2) | 1 | (2.17) |
| **Diaphragm defect size** | | | | |
| A | 13 | (4.89) | 2 | (4.35) |
| B | 67 | (25.2) | 5 | (10.9) |
| C | 73 | (27.4) | 21 | (46.7) |
| D | 18 | (6.77) | 3 | (6.52) |
| **Surgical procedure** | | | | |
| Primary closure | 54 | (20.3) | 6 | (13.0) |
| Patch closure | 182 | (68.4) | 34 | (73.9) |
| **Diuretics post surgery** | 201 | (75.6) | 32 | (69.6) |
| **Outcome data, *Means ± SD*** | | | | |
| Duration of MV[d] | 21.2 | ± 19.6 | 22.2 | ± 12.4 |
| Duration of VA [d] | 14.8 | ± 16.1 | 29.2 | ± 33.4 |
| CLD, *n (%)* | 105 | (39.5) | 26 | (56.6) |
| Duration of ECMO [d] | 9.77 | ± 4.20 | 8.91 | ± 3.57 |
| Duration of hospitalization [d] | 60.6 | ± 44.5 | 87.4 | ± 71.0 |
| Survival, *n (%)* | 219 | (82.3) | 39 | (84.8) |

CLD = chronic lung disease, ECMO = extracorporeal membrane oxygenation, FETO = fetoscopic tracheal occlusion, LCDH = left-sided congenital diaphragmatic hernia, MV = mechanical ventilation, o/e LHR = observed-to-expected lung-to-head-ratio, rFLV = relative fetal lung volume, RCDH = right-sided congenital diaphragmatic hernia, VA = ventilatory assist.
