# Supplementary material for: Pleural Effusion and Chylothorax in Congenital Diaphragmatic Hernia—Risk Factors, Management and Outcome
Source: J Clin Med. 2024 Mar 19;13(6):1764. doi: 10.3390/jcm13061764 (PMC10971182; doi:10.3390/jcm13061764)
Supplement: Supplementary file 1 [file jcm-13-01764-s001.zip › Supplemental Table S2.pdf]

Supplemental Table S2: Comparison of outcome parameters for chylothorax vs. no chylothorax

	Total		Chylothorax		No Chylothorax[a]		*p-value*
**Total count per group**	**312**	**(100)**	**58**	**(100)**	**221**	**(100)**	
o/e LHR [%]	40.3	± 13.7	37.7	± 13.7	41.1	± 13.6	0.14
rFLV [%]	36.8	± 16.4	30.6	± 11.1	38.7	± 17.4	< 0.001
5-minute APGAR	7.47	± 1.59	7.22	± 1.47	7.66	1.52	0.05
Gestational weight [g]	3061	± 506	2992	± 404	3087	± 538	-
ECMO prior to surgery, *n (%)*	137	(43.9)	39	(67.2)	78	(35.3)	<0.0001
PE post surgery, right [ml][b]	565	± 726	1112	± 882	201	± 169	0.03
PE post surgery, left [ml][c]	731	± 1229	1218	± 625	225	± 197	<0.0001
Duration of diuretics [d]	23.9	± 26.9	38.2	± 27.9	20.5	± 25.6	<0.0001
**Outcome parameter, *Means ± SD***							
Duration of MV [d]	21.3	± 18.7	34.9	± 24.0	18.8	± 16.1	<0.0001
Duration of VA [d]	17.2	± 20.5	26.6	± 20.2	15.1	± 20.1	0.0004
Pneumonia, *n (%)*	17	(5.45)	7	(12.1)	9	(4.07)	0.02
CLD, *n (%)*	131	(42.0)	47	(81.0)	80	(36.2)	<0.0001
Duration of ECMO [d]	9.57	± 4.05	10.8	± 4.53	8.73	± 3.41	0.01
Duration of hospitalization [d]	64.3	± 49.9	92.6	± 46.0	56.5	± 48.8	<0.0001
Survival, *n (%)*	258	(82.7)	48	(82.8)	199	(90.0)	0.12

CLD = chronic lung disease, ECMO = extracorporeal membrane oxygenation, MV = mechanical ventilation, o/e LHR = observed-to-expected lung-to-head ratio, PE = pleural effusion, rFLV = relative fetal lung volume, VA = ventilatory assist.

[a] includes all other CDH patients (including CDH patients without PE)

[b] right-sided PEs are composed of both cases of left- and right-sided CDH.

[c] left-sided PEs correspond to left-sided CDH only.
