# Supplementary material for: Pleural Effusion and Chylothorax in Congenital Diaphragmatic Hernia—Risk Factors, Management and Outcome
Source: J Clin Med. 2024 Mar 19;13(6):1764. doi: 10.3390/jcm13061764 (PMC10971182; doi:10.3390/jcm13061764)
Supplement: Supplementary file 1 [file jcm-13-01764-s001.zip › Supplemental Table S3.pdf]

Supplemental Table S3: Frequency of pleural effusion and drainage before and after surgery for left- and right-sided congenital diaphragmatic hernia

**LCDH (*n = 266*)**	***n, %***	***% of LCDHs***	**RCDH *(n=46)***	***n, %***	***% of RCDHs***
**Pleural effusion pre-surgery, *n (%)***	**48 (100)**	**(18.0)**	**Pleural effusion pre-surgery, *n (%)***	**12 (100)**	**(26.1)**
ipsilateral	18 (37.5)	(6.77)	ipsilateral	4 (33.3)	(8.70)
contralateral	20 (41.7)	(7.52)	contralateral	5 (41.7)	(10.9)
both sides	10 (20.8)	(3.76)	both sides	3 (25.0)	(6.52)
**Chest tube insertion pre-surgery**	**19 (39.6)**	**(7.14)**	**Chest tube insertion pre-surgery**	**5 (41.7)**	**(10.9)**
ipsilateral	5 (10.4)	(1.88)	ipsilateral	1 (8.33)	(2.17)
contralateral	13 (27.1)	(4.89)	contralateral	3 (25.0)	(6.52)
both sides	1 (2.08)	(0.38)	both sides	1 (8.33)	(2.17)
**Pleural effusion post-surgery, *n (%)***	**192 (100)**	**(72.2)**	**Pleural effusion post-surgery, *n (%)***	**31 (100)**	**(67.4)**
ipsilateral	165 (85.9)	(62.0)	ipsilateral	28 (90.3)	(60.9)
contralateral	2 (1.04)	(0.75)	contralateral	0 (0)	(0)
both sides	25 (13.0)	(9.40)	both sides	3 (9.68)	(6.52)
			n/a		
**Chest tube insertion post-surgery**	**113 (58.9)**	**(42.5)**	**Chest tube insertion post-surgery**	**18 (58.1)**	**(39.1)**
ipsilateral	105 (54.7)	(39.5)	ipsilateral	18 (58.1)	(39.1)
contralateral	2 (1.04)	(0.75)	contralateral	0 (0)	(0)
both sides	6 (3.13)	(2.26)	both sides	0 (0)	(0)

LCDH = left-sided congenital diaphragmatic hernia, RCDH = right-sided congenital diaphragmatic hernia
